# The Impact of School Opening Model on SARS-CoV-2 Community Incidence and Mortality: A Nationwide Cohort Study

**DOI:** 10.21203/rs.3.rs-712725/v1

**Published:** 2021-07-15

**Authors:** Zeynep Ertem, Elissa Schechter-Perkins, Emily Oster, Polly van den Berg, Isabella Epshtein, Nathorn Chaiyakunapruk, Fernando Wilson, Elli Perenchevich, Warren Pettey, Westyn Branch-Elliman, Richard Nelson

**Affiliations:** Binghampton University; Boston University Medical School; Brown University; Beth Israel Deaconess Medical Center; VA Boston Healthcare System; University of Utah; University of Utah; Center for Access & Delivery Research and Evaluation, Iowa City VA Health Care System, Iowa City, Iowa; IDEAS Center, Veterans Affairs Salt Lake City Healthcare System; VA Boston Healthcare System; University of Utah

**Keywords:** In-person Schooling, Remote Learning, Incidence Rates, Event Study

## Abstract

The role that in-person schooling contributes to community incidence of SARS-CoV-2 infections and deaths remains unknown. We conducted an event study evaluating the effect of in-person school on SARS-CoV-2 cases and deaths per 100,000 persons during the 12-weeks following school opening, stratified by US Census region.

There was no impact of in-person school opening and COVID-19 deaths. In most regions, COVID-19 incidence rates were not statistically different in counties with in-person versus remote school modes. However, in the South, there was a significant and sustained increase in cases per week among counties that opened for in-person learning versus remote learning, with weekly effects ranging from 7.8 (95% CI: 1.2–14.5) to 18.9 (95% CI: 7.9–29.9) additional cases per 100,000, driven by increases among 0–9 year olds and adults.

## Introduction

During the first few months of the COVID-19 pandemic, primary and secondary schools in the United States were closed to in-person education as part of the national response to control the spread of SARS-CoV-2.^1^ This decision was guided by data extrapolated from influenza transmission models, which suggested school closures as an effective measure for reducing the basic reproductive number of respiratory viral infections^1^ and early evidence suggesting that non-pharmaceutical public health interventions, including school closures, were associated with improved SARS-CoV-2 outbreak control.^2^

Modeling studies and time series analyses from across the world differ in their assessment of the impact of reopening schools on community SARS-CoV-2 transmission.^3–5^ Elementary-school aged children are at lower risk of severe illness than other age groups, and their role in transmission remains cloudy.^6,7^ However, there are multiple close interactions between individuals from separate households in a school setting, thus interactions that occur in schools, even if each contact is lower risk, may contribute to SARS-CoV-2 spread. If children and school staff become infected at school, these transmissions may lead to subsequent transmissions to family members and other contacts, potentially resulting in increases in community transmission of SARS-CoV-2. Recently published studies about the impact of school mode on community transmission from Indiana, Texas, and other states found conflicting results,^8–10^ with some analyses suggesting substantial increases in case rates associated with school openings, others suggesting a small impact, and still others suggesting that opening schools to in-person learning has no impact on community case rates.

Thus, the association between type of school reopening mode (e.g., virtual, hybrid, or in-person) and community spread of SARS-CoV-2 continues to be a critical policy question. Although school closure early in the pandemic was associated with lower COVID-19 incidence,^11^ the impact of school closures in addition to other public infection prevention measures, such as business restrictions, social distancing, masking, scale-up of testing and contact tracing remains unknown. Thus, the aim of this national, retrospective cohort study is to evaluate the impact of school mode and opening to in-person education on subsequent changes in community incidence of COVID-19.

## Results

**Figure 1:**
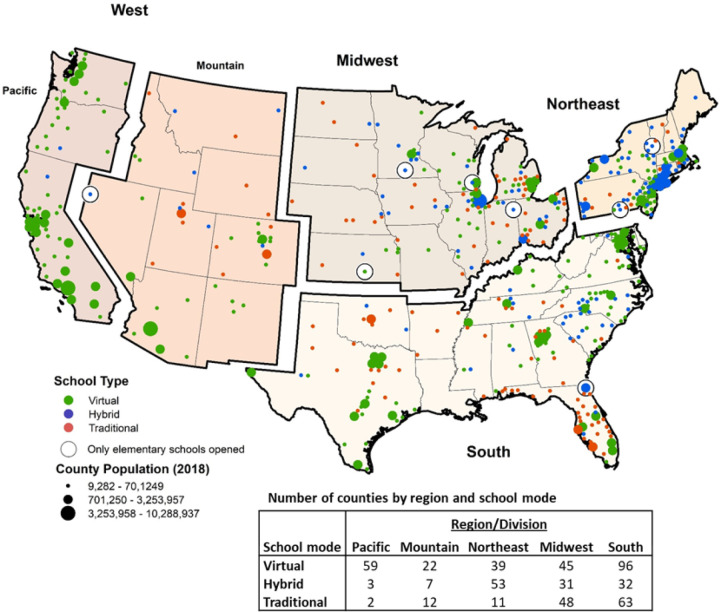
Map of counties included the analysis. Counties in green opened in a fully remote learning model, counties in blue opened in hybrid, and counties in red opened in a fully virtual learning mode. Size of county is correlated with county population size.

In total, 519 counties representing 1,050 school districts had school opening mode available. After excluding the Pacific region of the West due to limited variation (59/64 fully remote, 3 hybrid, 2 traditional), 459 counties comprised of 895 school districts were included (Figure 1). In all counties, one school mode predominated (i.e., there were no counties split evenly between remote and in-person learning).

Among the included counties, 103 were in the Northeast, 41 in the Mountain division, 124 in the Midwest, and 191 in the South (Table 1). Traditional, full in-person schooling was the most common mode in the Midwest (48/124); in the Northeast, hybrid learning models predominated (53/103); and in the South and Mountain division, virtual learning was the most common (South, 96/191, Mountain, 22/41).

Initial school opening dates varied but ranged from an earliest start date of 7/22/2020 to a latest start date of 9/28/2020 (Supplementary Figure 1). Notable demographic differences by region were identified (Table 1). Notable differences in community activity and infection control policies identified between regions include higher rates of business closure and activity restrictions in the Northeast and Western regions, increased contact tracing in the Northeast, stricter masking policies and regulations in the Northeast and West, and more access to testing in the Northeast and Midwest (Supplementary Table 1). Community-level mask mandates were generally implemented earlier in the Northeast versus other regions; masking mandates were least common and tended to be implemented latest in the South (5/20 Southern counties had no mask mandate or a masking mandate started after school opening versus 2/20 in the Midwest, 1/20 in the Northeast, and 0/20 in the West).

**Figure 2:**
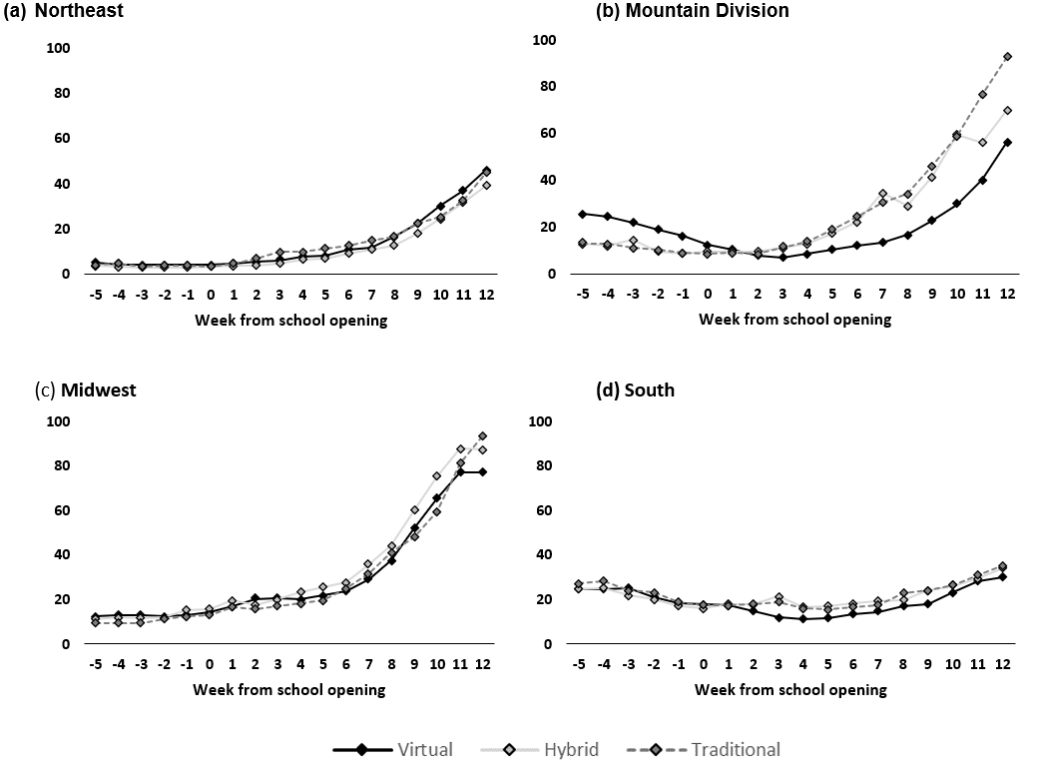
Map of counties included the analysis. Counties in green opened in a fully remote learning model, counties in blue opened in hybrid, and counties in red opened in a fully virtual learning mode. Size of county is correlated with county population size.

**Figure 3:**
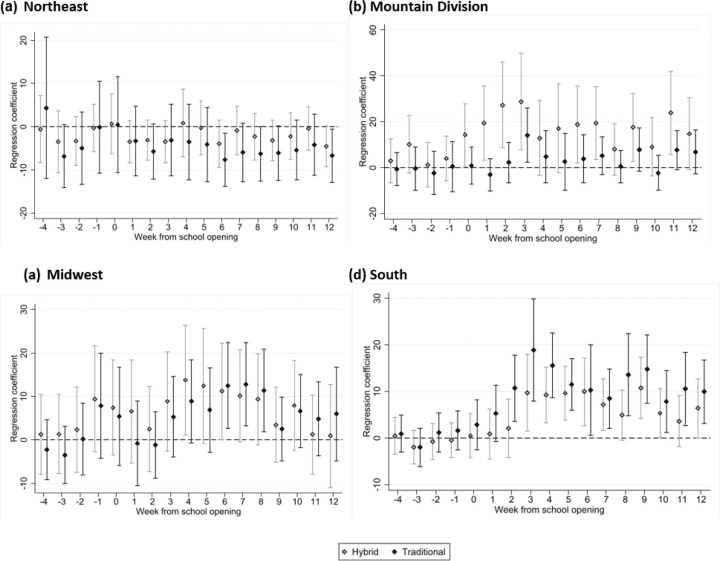
Unadjusted mean COVID deaths per 100,000 residents, stratified by region; week zero denotes initial school opening.

Unadjusted mean COVID-19 cases per 100,000 residents per week stratified by region are shown in Figure 2 and are notable for increases in COVID-19 case counts across all regions during the weeks following the start of school, regardless of school mode. The adjusted absolute differences in COVID-19 cases between counties with hybrid and traditional school opening modes relative to counties with virtual learning models are presented in Figure 3. In the Northeast, Mountain division, and Midwestern regions, differences in COVID-19 case rates were not detectably different across any of the three learning modes, although there was a small increase in cases 6–9 weeks following school opening in the Midwest among counties with traditional learning; no increase was found in counties with hybrid learning modes. In the South, there was a statistically significant increase in cases among counties that opened for hybrid or traditional modes when compared to virtual.

After adjustment, traditional school mode was associated with increases in the number of COVID-19 cases from week 6 compared to fully remote school mode (effect = 12.5 cases per 100,000 residents, 95% CI = 1.6–20.0) to week 8 (effect = 11.4, 95% CI = 1.9–20.8) in the Midwest. Similarly, in the South, traditional in-person mode was associated with increases in the number of COVID-19 cases during the period from week 2 following school opening (effect = 10.7 cases per 100,000 residents, 95% CI = 3.6–17.8) to week 12 following opening, (effect = 10.0, 95% CI = 3.1–16.8).

**Figure 4:**
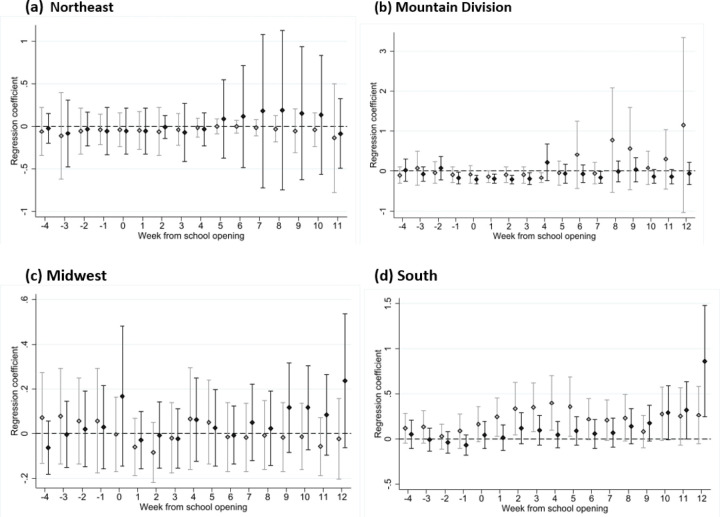
Adjusted absolute difference between COVID cases in counties with hybrid and traditional school modes relative to virtual for each week, with week 0 being the week in which school started for each county, stratified by region.

In the South, hybrid school mode was associated with increases in the number of COVID cases from week 3 (effect = 9.7, 95% CI = 1.5–17.9) to week 12 (effect = 6.4, 95% CI = 0.0–12.7); a similar trend was not found elsewhere. There was no impact of school opening mode on subsequent COVID-19 related deaths during the entire 12-week period following school opening in any region (Figure 4, Supplementary Figure 2).

In the adjusted models, the impact of school opening mode on COVID-19 cases stratified by age group varied between and within regions (Supplementary Figures 3–5). Across all regions, there were no differences in 10–19 year olds. Case increases associated with in-person learning in the South and Midwest were driven by increases in cases diagnosed in ≥20 year olds. In the South, there was a statistically significant increase in cases among 0–9 year-olds during the 2–10 week period following school opening.

## Discussion

This national cohort study, which included nearly half of all public student enrollment across the US, found regional variation on the impact of school re-opening policy on community incidence of SARS-CoV-2. In the South, which tended to have more limited community-level mitigation measures, re-opening schools for in-person learning (in either a hybrid or a traditional approach) was associated with a subsequent increase in community case rates of COVID-19, driven by case increases among adults and children under the age of 10. In the Midwest, opening in a traditional school mode was associated with increases in case counts. In other regions, where adoption of community public health measures tended to be higher, we found no impact of school opening mode on subsequent community incidence of SARS-CoV-2. These data add to a growing body of literature about the impact of school opening policy on SARS-CoV-2 transmission and public health measures for pandemic control.^20^

Although evidence demonstrates that children, particularly elementary school-aged children, are at very low risk of severe COVID-19,^21^ data are mixed about the role children may play in household and community transmission of SARS-CoV-2.^22,23^ Our nationwide study adds to a growing body of data about the role that in-person learning plays in SARS-CoV-2 transmission in the surrounding community and is consistent with prior studies supporting broad infection prevention strategies for SARS-CoV-2 control. Additionally, our study demonstrates that, while school opening can be associated with increases in case rates in some regions, those case increases may not translate to detectable increases in COVID-19 mortality. Thus, policy decisions to close schools must be weighed against the harms of ongoing school closures.

In our dataset, the most extreme increase associated with school openings was found in the South, where school opening was associated with a weekly increase in COVID cases ranging from 7.8 to 18.9 per 100,000 people. Notably, in the South, infection control measures both inside and outside of school were limited; in regions with more substantive infection control efforts both inside of school settings and in the broader communities, such as the Northeast, there was no increase in community case incidence associated with opening schools, and a trend toward a decrease in SARS-CoV-2 cases among children after schools opened for in-person instruction.

Our study adds to a growing body of literature about the impact of school closures as a policy measure for reducing the basic reproductive number of SARS-CoV-2. Early reports suggested that school closures were associated with reductions in COVID-19 deaths, although study authors acknowledged that, due to the simultaneous implementation of a variety of public health measures, the impact of school closure could not be fully delineated.^5,9–11^ Other investigations found conflicting results, with some suggesting that opening schools is associated with an increase in SARS-CoV-2 cases in the community and others suggesting minimal or no impact. A recent meta-analysis found that studies with the lowest risk of statistical bias did not find a substantial impact of school mode on community incidence.^3,20^ A recent study evaluating the impact of school mode on community SARS-CoV-2 cases in Texas found substantial increases associated with re-opening schools for in-person learning.^9^ However, the Texas study, unlike ours, did not control for temporal trends occurring prior to the start of school. Across all regions, school opening occurred in a background of increasing case counts, thus controlling for temporal trends and other county-level factors is critical for isolating the impact of school mode from other simultaneous events that may occur at the same time as changes in school learning modes.

Multiple prior studies have demonstrated low rates of transmission in urban, suburban, and rural public school settings, provided multifaceted infection prevention plans are implemented.^24–28^ Conversely, additional SARS-CoV-2 school clusters reported in the US and around the world highlight that substantial in-school transmission can occur, particularly in the absence of mitigation measures. This finding is supported by a recent national survey, which found that, among students attending schools that adopted few or no mitigation measures, living with a student attending in-person school was associated with a higher risk of COVID-19 among family members.^29^ However, this same study also found that the elevated risk was eliminated with the addition of more in-school mitigation measures, findings similar to others from around the world. ^5,30,31^

### Limitations:

Our study has several limitations. Detailed infection control plans were not available, thus we were not able to measure the effectiveness of specific infection prevention measures within schools on community incidence of cases. However, the aim of this study was to evaluate the impact of school opening policy on community transmission, not to address the related but distinct question of SARS-CoV-2 transmission and prevention in schools. We may not have been able to fully control for community infection prevention measures that may have impacted estimates. However, we attempted to mitigate the effect of this confounding by stratifying our analysis by region, which is highly correlated with school opening policy and state-level infection control interventions, and by using a variety of data sources to address community response in a variety of different ways, including both the Oxford dataset, which included detailed information about SARS-CoV-2 mitigation strategies and Google Movements data, which reflected county-level activity. The use of multiple robust datasets to control for these factors is a major strength. Data about school mode were available on a district level and other measures were available on a county or state level; it is possible that the conversion from district to county data may have introduced bias into our findings. However, Burbio’s validation data has found that school opening mode is highly clustered and estimate a margin of error of 2.7% in their dataset;^13^ this small margin of error would not have changed our study’s principal findings. Finally, we were not able to account for private schools; however, approximately 90% of elementary and secondary school-aged children attend public schools,^13^ thus the impact of this missing data is likely to be small.

## Conclusions

The impact of school opening mode on county incidence of SARS-CoV-2 varies by region and may be correlated with community infection prevention measures, which may also be correlated with implementation of in-school mitigation measures. These findings suggest that schools can open for in-person learning during the pandemic with minimal to no impact on community incidence of infections, provided other public safety measures are adopted.

## Methods

To measure the impact of school mode on community transmission, we created a retrospective cohort of school districts including the period immediately preceding and following school re-opening in the US (July-September, 2020, Supplementary Figure 1). Using multiple data sources, a longitudinal dataset at the county-week level was created and COVID-19 cases were examined. Variation in school opening date and mode were exploited to estimate the effect of initial school mode on COVID outcomes. Data spanning the time from 5 weeks before official school opening in any of three modes (e.g., traditional, hybrid, virtual) to 12 weeks after the start of school were included. County fixed effects were included to control for trends in case counts prior to school opening. In each of our statistical analyses, week 0 corresponds to the week in which school began in that county.

### Data Sources:

#### School Model:

School reopening mode data were obtained from Burbio, which includes manually-validated information from 1,200 school districts across the US, representing approximately 35,000 schools in 50 states, and 47% of student enrollment in public K-12 schools.^12,13^ Districts are classified into type of school mode, including *traditional*, defined as students participating in in-person learning ≥ four days per week; *hybrid*, defined as students divided into cohorts and attending school in-person two to three days per week; and *virtual*, defined as students attending school in a fully remote mode with no live, in-person instruction. Data available in the Burbio dataset include the date the school district opened and the proportion of schools that opened in each of the three different learning modes, stratified by school type (e.g., elementary, defined as kindergarten-5^th^ grade, middle school, defined as 6^th^-8^th^ grade, and high school, defined as 9^th^-12^th^ grade). To convert these school district-level data to the county level, we first took the average school mode proportion among sampled districts within a county across the 3 grade levels. We then assigned the school mode for the county based on the maximum value of these averaged grade level school modes; e.g., if 75% of the districts within a county were hybrid, then the entire county was considered hybrid.

#### Community Incidence and COVID-19-Related Deaths:

Incident cases of COVID-19 per day at a county level were obtained from the Centers for Disease Control and Prevention (CDC) dataset.^14^ Data available through these sources include daily cases, decade of age, and deaths by county, starting from January 2020.^4,15^ Per CDC guidelines, both confirmed and probable cases and deaths are included. Daily incidence was converted into a weekly incidence for cases and deaths. The denominator for the outcome measures is estimated number of residents in the year 2020 for each county by the US Census Bureau.

#### Community-Level COVID Mitigation Measures:

Data about community-level mitigation measures were obtained through the Oxford University dataset, which contain data about federal, state, and sub-state policies.^16,17^ To validate these data, a sample of districts (N=20 in each of the four census regions) underwent manual review for presence and type of community-level masking policy.

#### Community Mobility Data:

Community Mobility Data were accessed from the Google Movements dataset.^18^ These reports contain aggregated and anonymized user data through Google’s location history. The user data were organized into trends over time by geography, separated into various locations. The Community Mobility data provide insight into the mobility response to COVID-19 mitigation policies.^18^ These variables are measured as the percentage change in the time individuals spent in different locations relative to a baseline time period (1/3/2020–2/6/2020).

#### Independent variables:

The key independent variables were the county school mode, dummy variables for each week, and the interaction between the school mode and week variables. Our analyses controlled for important co-variates to minimize confounding bias in the relationship between school mode opening and the outcomes. These co-variates included variables from the Google mobility data (retail and recreation, grocery and pharmacy, workplaces, and residential) and from the Oxford policy data (workplace closings, canceling of public events, restrictions on gatherings, closing of public transportation, COVID testing policies, COVID contact tracing, and requirements to wear masks outside of the home). In addition, we included county, state, week, and state-week fixed effects to control for temporal trends, among other county-level factors. Given regional variation and correlation within regions regarding SARS-CoV-2 case counts, county infection control strategies, and school mode, the cohort was stratified by US census region (e.g., Northeast, West, Midwest, South). The Pacific Division was excluded due to near uniform school mode (virtual); the West region, therefore, includes only the Mountain Division.

#### Outcome variables:

The primary outcome variable was change in county-level incidence of COVID-19 diagnoses per 100,000 residents. Secondary outcome variables included change in COVID-19 mortality per 100,000 residents and the change in incident diagnoses stratified by decade of life (0–9 years, 10–19 years, and 20+ years), which was examined to determine if school model was associated with increases in children and adolescents attending primary and secondary school, or if the primary impact was on infections diagnosed in adults.

### Data Analysis:

We used an event-study framework^19^ with data from before and after K-12 schools opened for the 2020–2021 school year. We estimated the effect of school opening mode on COVID-19 diagnoses and mortality outcomes using a multivariable Poisson regression with robust standard errors. We ran models separately for each of the 4 regions. We report results from these models from the school mode-week interaction terms as marginal effects which are interpreted as the adjusted absolute effect of school mode per week on the outcome. All analyses were completed using STATA version 16.

## Supplementary Material

Supplement 1

## Figures and Tables

**Table 1:** Descriptive statistics of independent variables at county level on the week of school opening

	Northeast		Mountain Division		Midwest		South		
Characteristics	N/Mean	%/SD	N/Mean	%/SD	N/Mean	%/SD	N/Mean	%/SD	P-value
**Total**	103		41		124		191		
**Race/Ethnicity**									
% White	82.5%	11.8%	83.4%	10.8%	84.0%	9.0%	73.2%	15.1%	<0.001
% Black	7.6%	7.2%	2.2%	2.7%	8.0%	7.2%	17.5%	14.0%	<0.001
% Other	9.9%	6.3%	14.4%	9.9%	8.0%	3.9%	9.3%	5.7%	<0.001
% Hispanic	9.6%	8.4%	21.2%	14.3%	7.2%	7.0%	13.9%	15.5%	<0.001
**Median income (mean)**	$74,066	17,572	$68,781	14,827	$64,344	13,466	$66,398	18,596	<0.001
**Urban**	46	44.7%	8	19.5%	40	32.3%	82	42.9%	0.009

## Data Availability

Data underlying this manuscript were collected from a variety of sources. Community Mobility Report data from Google and the COVID-19 Government Response Tracker data from Oxford University are publicly available for download. Data from Burbio, LLC are available via purchase agreement and data use agreement with the company. Data from the CDC restricted access dataset are available via data use agreement with the CDC.
