# Supplementary material for: The Impact of School Opening Model on SARS-CoV-2 Community Incidence and Mortality: A Nationwide Cohort Study
Source: Res Sq. 2021 Jul 15:rs.3.rs-712725. Preprint. [Version 1] doi: 10.21203/rs.3.rs-712725/v1 (PMC8288150; doi:10.21203/rs.3.rs-712725/v1)

Supplementary Figures and Tables.

**Supplementary Table 1:** Descriptive statistics of covariates included in regression models

|  | **Northeast** | | **Mountain West** | | **Midwest** | | **South** | |  |
| --- | --- | --- | --- | --- | --- | --- | --- | --- | --- |
| **Characteristics** | **N/Mean** | **%/SD** | **N/Mean** | **%/SD** | **N/Mean** | **%/SD** | **N/Mean** | **%/SD** | **P-value** |
| **Total** | 103 |  | 41 |  | 124 |  | 191 |  |  |
| **Google movement data (mean % change from baseline)** |  |  |  |  |  |  |  |  |  |
| Retail and recreation | -7.84 | 23.27 | -6.58 | 12.77 | -5.18 | 9.85 | -11.73 | 14.78 | <0.001 |
| Grocery and pharmacy | 2.75 | 14.36 | 7.74 | 13.15 | 6.19 | 10.72 | -1.72 | 13.60 | <0.001 |
| Workplaces | -29.54 | 7.09 | -29.38 | 5.96 | -27.43 | 6.01 | -31.00 | 6.54 | <0.001 |
| Residential | 7.78 | 2.59 | 7.17 | 3.00 | 6.23 | 2.56 | 9.21 | 2.93 | <0.001 |
| **Oxford policy variables** |  |  |  |  |  |  |  |  |  |
| *Workplace closing* |  |  |  |  |  |  |  |  |  |
| No measures | 0 | 0.0% | 0 | 0.0% | 0 | 0.0% | 0 | 0.0% | <0.001 |
| Recommend closing | 8 | 7.8% | 8 | 19.5% | 79 | 63.7% | 79 | 41.4% |  |
| Require closing for some sectors | 95 | 92.2% | 33 | 80.5% | 45 | 36.3% | 112 | 58.6% |  |
| Require closing for all sectors but essential workplaces | 0 | 0.0% | 0 | 0.0% | 0 | 0.0% | 0 | 0.0% |  |
| *Cancelation of public events* |  |  |  |  |  |  |  |  |  |
| No measures | 72 | 69.9% | 22 | 53.7% | 66 | 53.2% | 78 | 40.8% | <0.001 |
| Recommend cancelling | 0 | 0.0% | 0 | 0.0% | 0 | 0.0% | 0 | 0.0% |  |
| Require cancelling | 31 | 30.1% | 19 | 46.3% | 58 | 46.8% | 113 | 59.2% |  |
| *Restrictions on gatherings* |  |  |  |  |  |  |  |  |  |
| No restrictions on gatherings | 3 | 2.9% | 6 | 14.6% | 15 | 12.1% | 14 | 7.3% | <0.001 |
| Restrictions on gatherings of >1000 people | 0 | 0.0% | 0 | 0.0% | 24 | 19.4% | 0 | 0.0% |  |
| Restrictions on gatherings of 101-1000 people | 0 | 0.0% | 2 | 4.9% | 9 | 7.3% | 9 | 4.7% |  |
| Restrictions on gatherings of 11-100 people | 100 | 97.1% | 16 | 39.0% | 42 | 33.9% | 86 | 45.0% |  |
| Restrictions on gatherings of <11 people | 0 | 0.0% | 17 | 41.5% | 34 | 27.4% | 82 | 42.9% |  |
| *Closing public transportation* |  |  |  |  |  |  |  |  |  |
| No measures | 24 | 23.3% | 19 | 46.3% | 70 | 56.5% | 75 | 39.3% | <0.001 |
| Recommend closing | 76 | 73.8% | 16 | 39.0% | 54 | 43.5% | 110 | 57.6% |  |
| Require closing | 3 | 2.9% | 6 | 14.6% | 0 | 0.0% | 6 | 3.1% |  |
| *Testing policies* |  |  |  |  |  |  |  |  |  |
| No testing policy | 0 | 0.0% | 0 | 0.0% | 0 | 0.0% | 0 | 0.0% | <0.001 |
| Only those with symptoms & meet specific criteria | 0 | 0.0% | 0 | 0.0% | 0 | 0.0% | 11 | 5.8% |  |
| Testing of anyone showing Covid-19 symptoms | 25 | 24.3% | 33 | 80.5% | 28 | 22.6% | 101 | 52.9% |  |
| Open public testing | 78 | 75.7% | 8 | 19.5% | 96 | 77.4% | 79 | 41.4% |  |
| *Contact tracing* |  |  |  |  |  |  |  |  |  |
| No contact tracing | 26 | 25.2% | 16 | 39.0% | 72 | 58.1% | 119 | 62.3% | <0.001 |
| Limited contact tracing | 0 | 0.0% | 0 | 0.0% | 0 | 0.0% | 0 | 0.0% |  |
| Comprehensive contact tracing | 77 | 74.8% | 25 | 61.0% | 52 | 41.9% | 72 | 37.7% |  |
| *Facial coverings* |  |  |  |  |  |  |  |  |  |
| No policy | 0 | 0.0% | 0 | 0.0% | 13 | 10.5% | 22 | 11.5% | <0.001 |
| Recommended | 0 | 0.0% | 0 | 0.0% | 0 | 0.0% | 0 | 0.0% |  |
| Required in some shared/public spaces | 6 | 5.8% | 19 | 46.3% | 40 | 32.3% | 23 | 12.0% |  |
| Required in all shared/public spaces | 72 | 69.9% | 22 | 53.7% | 71 | 57.3% | 122 | 63.9% |  |
| Required outside the home at all times | 25 | 24.3% | 0 | 0.0% | 0 | 0.0% | 24 | 12.6% |  |

**Supplementary Figure 1.** School Start Dates by region and mode

1. **Northeast (b) Mountain Division**

**(c) Midwest (d) South**

**Supplementary Figure 2**: Unadjusted mean COVID deaths per 100,000 residents

1. **Northeast (b) Mountain Division**

(c) **Midwest (d) South**


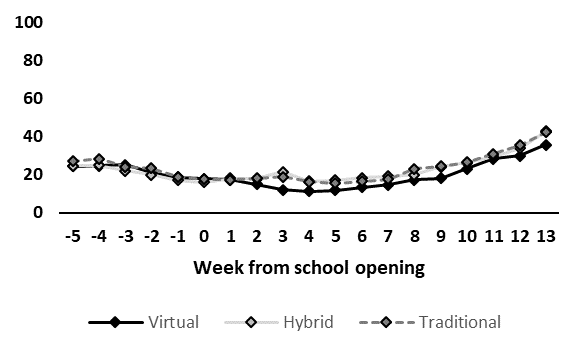


**Supplementary Figure 3**: Multivariable regression coefficients – COVID cases – ages 0-9

1. **Northeast (b) Mountain Division**


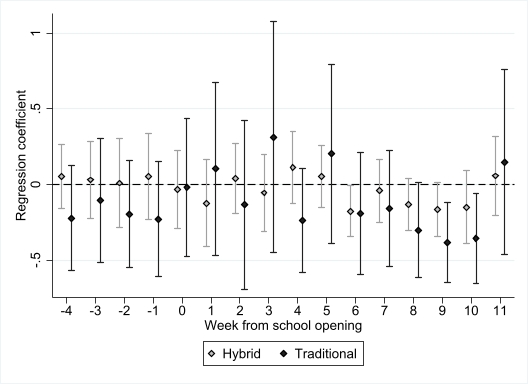

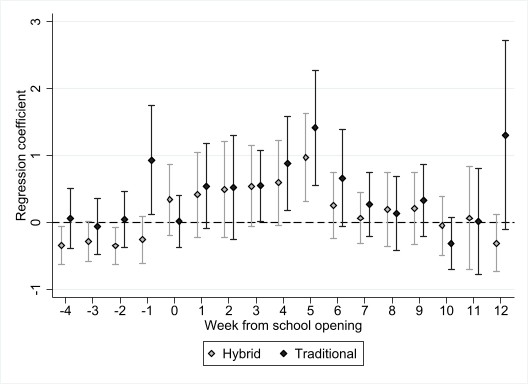


1. **Midwest (d) South**


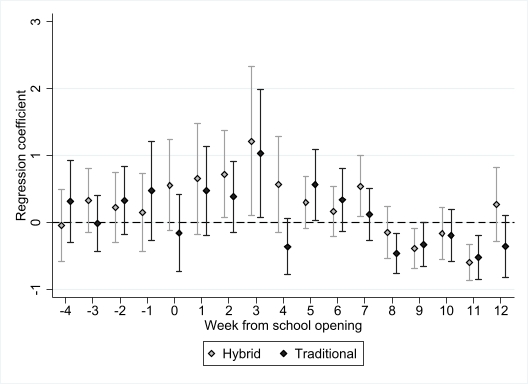

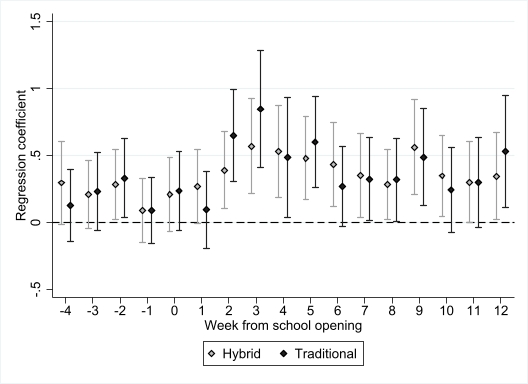


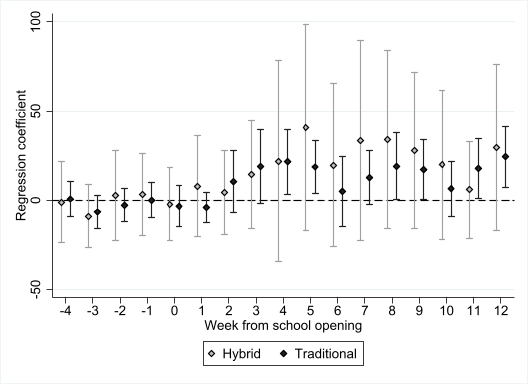


**Supplementary Figure 4**: Multivariable regression coefficients – COVID cases – ages 10-19

1. **Northeast (b) Mountain Division**


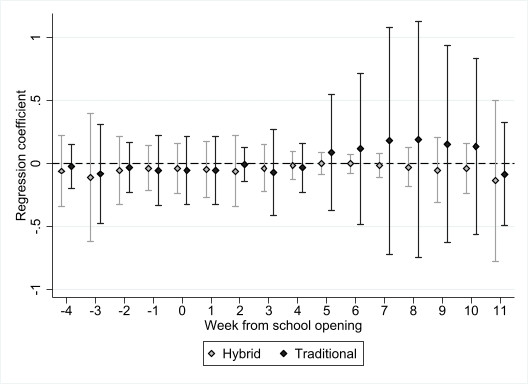

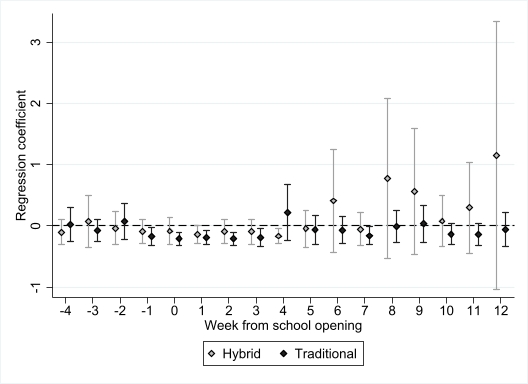


**(c) Midwest (d) South**


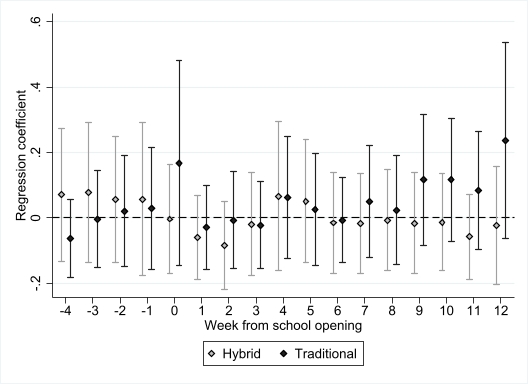

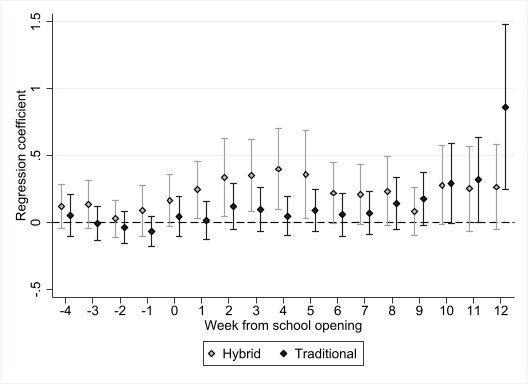


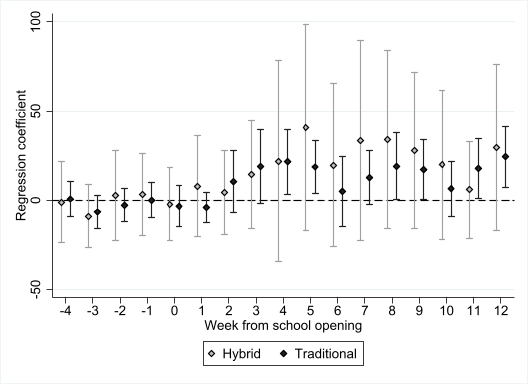


**Supplementary Figure 5**: Multivariable regression coefficients – COVID cases – ages 20+

1. **Northeast (b) Mountain Division**


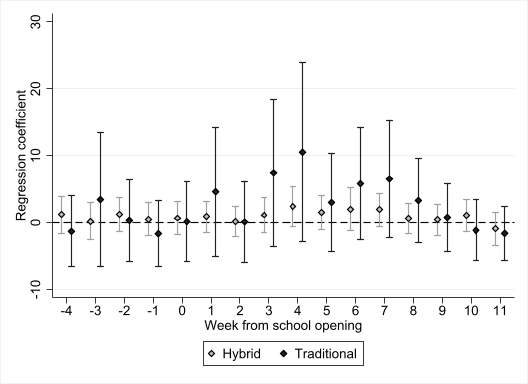

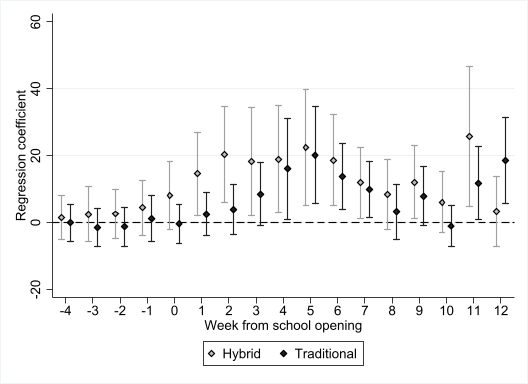


**(c) Midwest (d) South**


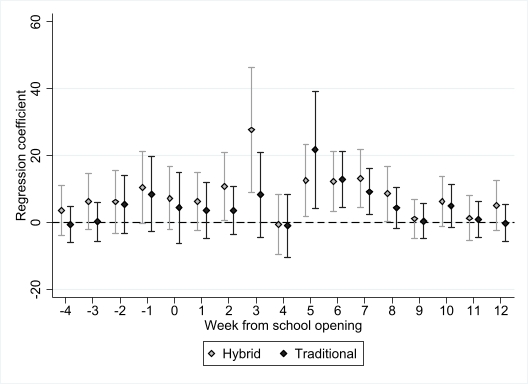

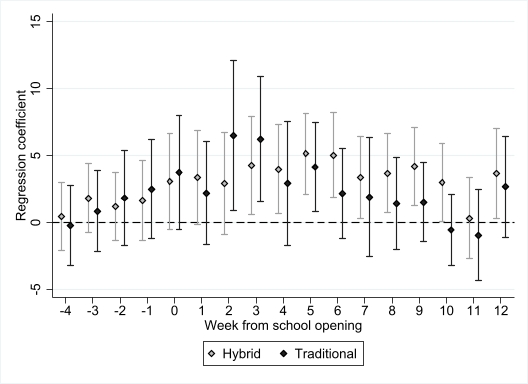


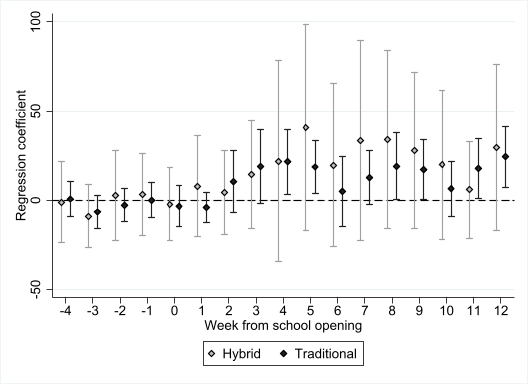

Supplement: Supplement 1 [file 94053520c193ec8397f0cbe7.docx]
